# A quality optimization approach to image Achilles tendon microstructure by phase-contrast enhanced synchrotron micro-tomography

**DOI:** 10.1038/s41598-021-96589-w

**Published:** 2021-08-27

**Authors:** Maria Pierantoni, Isabella Silva Barreto, Malin Hammerman, Lissa Verhoeven, Elin Törnquist, Vladimir Novak, Rajmund Mokso, Pernilla Eliasson, Hanna Isaksson

**Affiliations:** 1 Department of Biomedical Engineering, Lund University, Box 118, 221 00 Lund, Sweden; 2 Department of Biomedical and Clinical Sciences, Linköping University, 581 83 Linköping, Sweden; 3 Swiss Light Source, Paul Scherrer Institute, 5232 Villigen, Switzerland; 4 Division of Solid Mechanics, Lund University, Box 118, 221 00 Lund, Sweden

**Keywords:** X-ray tomography, Tendons

## Abstract

Achilles tendons are mechanosensitive, and their complex hierarchical structure is in part the result of the mechanical stimulation conveyed by the muscles. To fully understand how their microstructure responds to mechanical loading a non-invasive approach for 3D high resolution imaging suitable for soft tissue is required. Here we propose a protocol that can capture the complex 3D organization of the Achilles tendon microstructure, using phase-contrast enhanced synchrotron micro-tomography (SR-PhC-μCT). We investigate the effects that sample preparation and imaging conditions have on the resulting image quality, by considering four types of sample preparations and two imaging setups (sub-micrometric and micrometric final pixel sizes). The image quality is assessed using four quantitative parameters. The results show that for studying tendon collagen fibers, conventional invasive sample preparations such as fixation and embedding are not necessary or advantageous. Instead, fresh frozen samples result in high-quality images that capture the complex 3D organization of tendon fibers in conditions as close as possible to natural. The comprehensive nature of this innovative study by SR-PhC-μCT breaks ground for future studies of soft complex biological tissue in 3D with high resolution in close to natural conditions, which could be further used for in situ characterization of how soft tissue responds to mechanical stimuli on a microscopic level.

## Introduction

Tendons connect muscles to bones and transmit forces to translate muscle contractions into joint movements. The Achilles tendon connects the calf muscle to the heel bone and is the largest tendon in the human body. It is also the most frequently injured tendon^1^. The Achilles tendon is composed of hierarchically arranged collagen fibres, a non-collagenous matrix, and water^2^. The collagen assembles in fibrils which arrange to form fibers that build up the tendon fascicles^3^. The properties of the tendons are determined by the composition and organization of the collagen at each structural level^4^. The highly organized multi-level structure results in a tissue with rather high tensile strength^5,6^. The Achilles tendon is mechanosensitive and actively adapts to its mechanical loading environment^7–9^. However, the full correlation between the mechanical stimuli and the resulting complex 3D organization of the collagen is yet to be elucidated. To unravel this relationship, well-established high-resolution 3D imaging techniques, suitable for imaging soft biological tissues, such as the tendon, without affecting the mechanical properties are warranted.

Currently, most structural studies of tendon tissue are based on histology of thin sections in combination with chemical staining and/or immunohistochemistry^10,11^. Histology is highly informative, but it remains a destructive 2D technique^12^. Serial sectioning can be performed to study the structure in 3D, but this approach is very time consuming and can infer artefacts through sample preparation^13,14^. 3D visualization of tendons has been obtained at sub-micrometric resolution by combining focused ion beam milling with scanning electron microscopy^15^. However, this technique is also destructive and can only be performed on very small volumes (conventional field of view < 100 × 100 µm). Confocal or multiphoton microscopy can represent a good alternative for 3D visualization with sub-micrometric resolution^16,17^. However, the penetration depth is limited (typically < 500 µm) limiting the thickness of the samples that can be studied. Magnetic resonance imaging is also used for 3D acquisitions, but this approach is limited by its resolution (commonly > 100 μm)^18–20^.

Synchrotron X-ray tomography is a non-destructive method for 3D visualization and quantitative analysis of materials^21^. It allows to quickly obtain sub-micrometre resolution images, of relatively large volumes (~ 1 to 200 mm^3^ depending on the desired magnification), without invasive or time-consuming sample preparation. Furthermore, the images acquired by synchrotron X-ray tomography have isotropic resolution and can be virtually sliced in all directions with sub-micrometric precision. In clinical and conventional X-ray tomography the image contrast is proportional to the difference in X-ray attenuation between the materials composing the object. High contrast is achieved only between two regions of the object with sufficiently different X-ray attenuation coefficients^22^. Thus, due to the low absorption and small density differences, imaging soft biological materials is very challenging. Phase-contrast enhanced synchrotron micro-tomography (SR-PhC-μCT) was developed more recently to differentiate between sample regions that weakly absorb or have similar electron densities^23,24^. The contrast is amplified by the wave propagation phenomena, where the wave front is perturbed according to the variation of the refractive index of the materials present in the sample^25^. The perturbed wavefronts, when allowed to interfere in the space between the sample and the detector, generate a structural pattern and enhance the image contrast compared to pure attenuation images^26,27^. Examples of recent SR-PhC-μCT studies of biological tissues include e.g. muscles, heart, brain, lungs, ligaments, vessels, bone-soft tissue interfaces, intervertebral discs, and nerves^28–39^. One recent study has also shown the potential of SR-PhC-μCT to investigate the tendon structure. However, in this study the samples were prepared by cell maceration which leaves the tissue in a far from native condition^40^. Consequently SR-PhC-μCT has not yet been exploited to fully describe the complex Achilles tendon microstructure in a close-to-native state.

Recent studies have shown the importance of systematically studying the effect of different types of sample preparation protocols and imaging conditions to best describe the inner structure of complex soft biological tissues^21,33,41^. Fresh frozen samples imaged in saline solution enable conditions close to native. However, the samples can be unstable, where e.g. dehydration from the beam during imaging and sample micro-movements can reduce the image quality^33^. Fixation of the tissue can increase stability, but the images could still be affected by vibrations^41^. Samples embedded in solid media are generally more stable during imaging, but they require more invasive preparation. Furthermore, the tissue contrast could be reduced by the embedding medium. However, it should be noted that in many of the mentioned studies, where the effect of the sample preparation was evaluated, the final image quality was still determined subjectively^33,42^. The human eye indeed provides a good initial assessment of image quality, but because of the subjective nature, the outcome can vary substantially depending on who is evaluating the images. As shown recently, an objective analysis, as automatic as possible, is needed to limit the bias and to quantitatively assess image quality^43–46^. For instance, a recent study evaluated the quality of images acquired with synchrotron radiation computed tomography on breast tissue and compared the image quality to a clinical computed tomography images of the breast. In this study contrast-to-noise ratio, signal-to-noise ratio, noise power spectrum, spatial resolution and detail visibility were considered^46^.

This study aims to determine if SR-PhC-μCT is a suitable technique to study collagen fibers in Achilles tendons, and to define how different sample preparations and imaging set ups affect the resulting image quality. As such, we pave the ground for future high-resolution structural studies of tendon fibers and other complex soft biological tissues. Specifically, we compared fresh frozen samples in different solutions, fixed samples and samples embedded in paraffin. We also tested two different imaging setups with different final resolutions. Furthermore, quantitative parameters that together provide an objective characterization of the final image quality are presented.

## Methods

### Animal model

Eighteen female Sprague-Dawley rats (12 weeks old) were used for this experiment. The rats were kept two per cage with temperature maintained at 21 °C and a 12-h light/dark cycle. Rats were given food and water ad libitum. The rats were euthanized with carbon dioxide and the left Achilles tendon was harvested together with the calcaneal bone and the gastrocnemius-soleus muscle complex. The tendons were kept moist in phosphate-buffered saline solution (PBS) and then frozen at − 20 °C (eight tendons) or fixed (ten tendons). The experiments adhered to the institutional guidelines for the care and treatment of laboratory animals. The study was approved by the Regional Ethics Committee for animal experiments in Linköping, Sweden (Jordbruksverket, ID1424), and was performed in compliance with the ARRIVE Guidelines.

### Sample preparation and sample mounting

**Figure 1:**
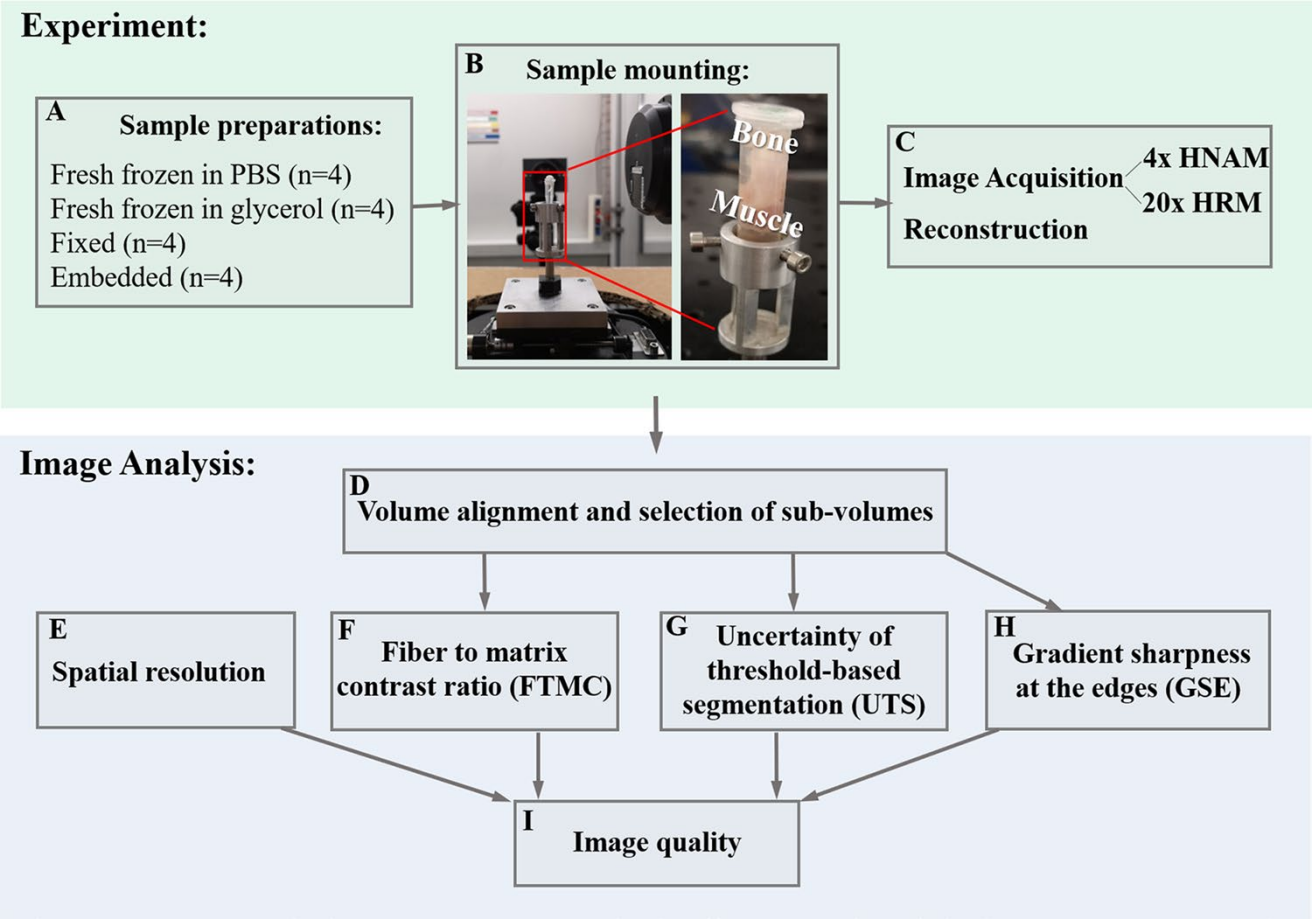
Schematic of the overall study. (**A**) 16 tendons were divided into four types of sample preparations. (**B**) The samples were mounted in Eppendorf tubes with the bone on the upper side and the muscle in the tube tip. The tubes were carefully fixed into the sample holder. (**C**) Imaging of the samples were performed using two different microscope setups. (**D**) The volumes were aligned, and sub-volumes were selected for analysis. (**E**–**H**) Image analysis determined four parameters. The four parameters were used to determine overall image quality (**I**).

Four types of sample preparations were evaluated: fresh frozen tendons in PBS solution, fresh frozen tendons in glycerol, fixed tendons in ethanol, and fixed tendons embedded in paraffin, all with n = 4 per preparation type (Fig. 1A). Fresh frozen tendons were stored at − 20 °C until the mounting when half were immersed in PBS and half in glycerol. Fixed tendons were left in 4% formaldehyde at room temperature for 48 h, then washed and stored in 70% ethanol at 4 °C. The tendons that were prepared for paraffin embedding, were first fixed in formaldehyde, and were then dehydrated in ethanol and embedded in paraffin. The paraffin blocks were carefully cut about 1 mm from the tendon, ensuring that no cracks were formed in the region of interest. Fresh and fixed tendons were mounted in 2 ml Eppendorf tubes filled with PBS or glycerol and ethanol respectively. The calcaneal bone, at the tendon-bone junction, was glued to the lid, then the tendon was gently pushed into the tube ensuring it was straight, the muscles were pressed into the tip of the tube until they were blocked, and no more movements of the tissue were allowed. All visible bubbles were removed by suction using a syringe. The Eppendorf tubes were mounted on a sample holder at the beamline (Fig. 1B). The tubes were oriented such that the bone junction was up, and the muscles were down. The paraffin blocks were glued to the stage holder using hot glue.

### Phase contrast enhanced synchrotron X-ray micro-tomography (SR-PhC-μCT)

The experiment was carried out at the X02DA TOMCAT beamline at the Swiss Light Source (SLS), Paul Scherrer Institute (Villigen, Switzerland)^47^. The tomographic setup was optimized to acquire phase contrast images for single distance phase retrieval. As the pixel size δ is given by the details to be resolved in the sample, the X-ray wavelength, λ by sample transmission, the sample to detector distance (z_D_) remains to be optimized. An initial value is typically given by the size of the first interference fringe as z_D_ = (2δ)^2^/λ. This parameter was optimized for each one of the two optical microscope setups for X-ray to visible light conversion that were used. The two optical microscope setups were (Fig. 1C).

- *High Numerical Aperture Microscope (HNAM)* (custom made objective, optiquepeter.com), 4× magnification, field of view (FOV) of 4.2 mm × 3.5 mm and final voxel size of 1.63 µm × 1.63 µm × 1.63 µm). The optical setup was coupled to a LuAG:Ce scintillator screen of 150 μm. The optical setup was coupled to a LuAG:Ce scintillator screen of 150 μm. For HNAM, the theoretically maximal value of the sample to detector distance to fulfil the single distance phase retrieval would be ~ 130 mm. However, our optimization procedure converged to a larger distance of 150 mm for which we achieved the best compromise between contrast and resolution after tomographic reconstruction. To visualize the subtle contrast between the fibers and the matrix the X-ray energy was set at 15 keV at which the X-ray matter interaction in our system was sufficiently strong but still allowed transmission (20–40%) through the sample. 2001 projections were acquired over 180° of continuous rotation. Projections were collected by a pco.edge 5.5 Camera (detector sCMOS sensor of 2560 × 2160 pixels, 6.5 μm pixel size and a 16-bit nominal dynamic range). The exposure time was 33 ms resulting in about 90 s scans and the skin dose for a single projection about 6 Gy. The HNAM setup was used to assess all four sample preparation protocols to investigate their effect.
- *High resolution microscope (HRM)* (objective UPLAPO20x, 20× magnification, FOV of 0.7 mm × 0.8 mm and final voxel size 0.33 µm × 0.33 µm × 0.33 µm). HRM was coupled to a LuAG:Ce scintillator screen of 20 μm. For HRM, the theoretical sample to detector distance would be ~ 5 mm. As for HNAM, our optimization resulted again in a larger value of 54 mm. This time the difference to the theoretical value is rather large, in part due to the fact that the true resolution of this optical setup is coarser than 2δ. The beam energy was set to 15 keV, and 4001 projections were acquired over 360° continuous rotation. Projections were collected by a pco.edge 5.5 Camera (detector sCMOS sensor of 2560 × 2160 pixels, 6.5 μm pixel size and a 16-bit nominal dynamic range). The exposure time was 100 ms, resulting in about 8 min scans. We calculated the skin dose rate to be 200 Gy/s^48^ therefore during a single radiographic projection the skin dose at the sample is 20 Gy. The actual dose which the sample receives is smaller due to its finite size, but the skin dose is a good measure of the upper limit. This setup was used to test how an increase of magnification would affect the image quality, and fresh frozen samples in PBS and samples fixed in ethanol were imaged.

#### Imaging and volume reconstructions

**Figure 2:**
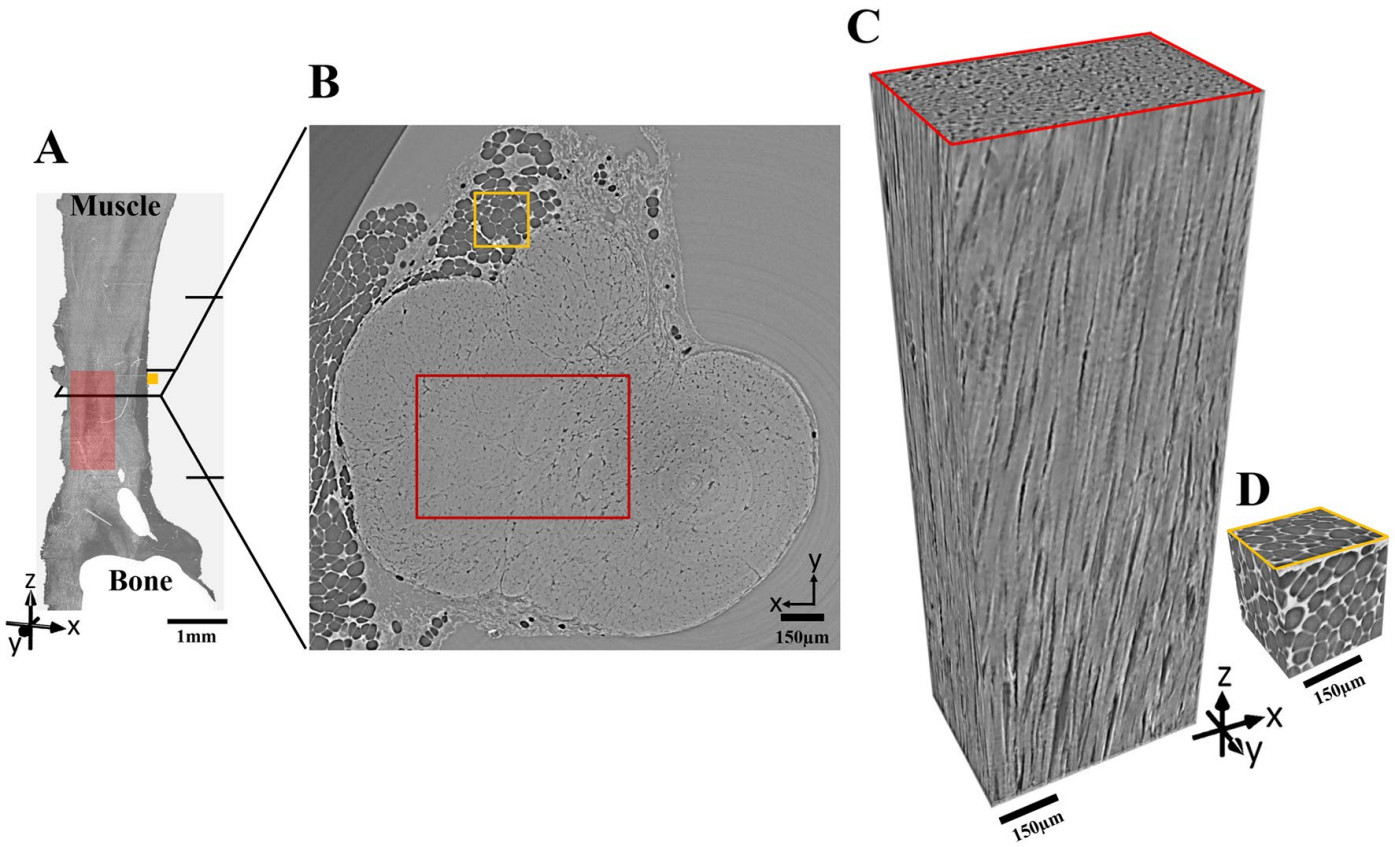
The regions of interest selected for the analysis of image quality. (**A**) volume rendering of the entire imaged tendon after reorientation and stitching of three consecutive the image stacks using the HNAM setup (the field of view of each scan is indicated by the black horizontal lines on the right side); (**B**) cross section of the tendon in the central region indicated in (**A**) by the black rectangle. The red and yellow squares in (**A**) and (**B**) indicate the regions chosen for analysis. (**C**) Volume rendering of the region highlighted in red showing the fiber organization; (**D**) volume rendering of the region highlighted in yellow showing the 3D arrangement of the fat cells that surround the tendon.

All samples were imaged with HNAM in consecutive locations from the muscles to the bone moving the FOV vertically 3 mm each time, this allowed to image the tendon from the muscle junction to the bone junction (Fig. 2A). Three fresh and three fixed samples were also imaged with HRM. In this case, the tendon was imaged with 4–6 consecutive vertical scans from the muscles to the bone moving the FOV vertically 0.6 mm each time. All projections were corrected with dark and flat-field images. The projected density of the sample was calculated using the Paganin phase retrieval for homogeneous objects^49^. The tomographic reconstruction was performed using a Fourier based regridding algorithm^50^. We used the following procedure to optimize the ratio of δ:β required for the reconstructions:

- the absorption coefficient β = 9.3 × 10^–9^ was determined for tendon tissue using the X-ray mass attenuation coefficients and density for soft tissues (ICRU-44) according to the NIST database^51^.
- The refractive index decrement δ was varied between 10:1, 50:1 and 100:1, by increasing the contribution of the phase retrieval.
- The intensity profile over 200 pixels was determined on the same location. Based on the intensity profile reconstructions were performed using a ratio of 50:1 (Supplementary Fig. S1). In fact, the interference fringe is visible when using a 10:1 ratio (Supplementary Fig. S1A). When using a 100:1 ratio, the small features are lost (Supplementary Fig. S1C). Finally, using a 50:1 ratio it is still possible to distinguish all features, but the fringe is filtered (Supplementary Fig. S1B).

For each microscope setup all data were reconstructed using the same gray scale intervals.

### Image quality analysis

Each volume was reoriented in ImageJ^52^, so that the tendon main axis was parallel to the z direction (Figs. 1D and 2A,B). Volume rendering and videos were created with Dragonfly (v 4.1, ORS software)^53^.

For the image quality analysis, sub-volumes of ~ 0.3–0.4 mm^3^ for HNAM and of ~ 0.02–0.03 mm^3^ for HRM containing only fibers were chosen in the middle of the tendons. Example sub-volumes are shown in Fig. 2C and Supplementary video S1. Smaller cubic sub-volumes of 0.015 mm^3^ containing only fat cells were also chosen to evaluate image sharpness (Fig. 2D). Four main properties (Fig. 1E–H) were calculated to determine the images quality using custom made MATLAB scripts.

- *Spatial resolution* The resolution was determined using a criterion based on the Power spectrum (Fourier domain) analysis^54^. This method reflects similar image properties (noise content) as the Fourier Shell Correlation approach which is also often used in tomography. Line profiles were taken in x and y directions (see coordinate system in Fig. 2C) and the power spectral density was calculated as described by Lovric et al.^55^. From the fiber sub-volumes, three slices along the z direction were considered, at the top, in the middle and at the end of the sub-volume. The spectral density converged at the level of the noise. The final spatial resolution was obtained by considering twice the value at converge and by coupling it to the respective spatial frequency.
- *Fiber-to-matrix contrast ratio (FMCR)* By considering the fibers as the material of interest and the matrix around them as the background, the FMCR corresponds to the contrast-to-noise ratio (Fig. 1F). It was calculated as described by Saccomano et al. and Lovric et al.^21,55^, using the following equation:
  1 $$FMCR=\frac{Im_{Fibers} - Im_{Matrix}}{\sqrt{\frac{1}{2}({\sigma }^{2}_{Fibers}+ {\sigma }^{2}_{Matrix} )}}$$
  where Im is the mean voxel value and σ is the standard deviation for fibers and matrix, respectively.
- *Uncertainty of the threshold-based segmentation of fibers and matrix (UTS)* For the exploitation of images in terms of the scientific results perhaps the most important measure of image quality is how reliably one can segment the structure of interest from the images. It is not easy to express this reliability. We proposed to quantify the relative number of voxels with gray scale values which did not allow to systematically differentiate if they belonged to the fiber or the matrix phase (Supplementary Fig. S2A). To calculate the UTS, we found the threshold value for which the fibers are selected as precisely as possible and the value to precisely select the matrix. To minimize bias, the value was selected using the Otsu’s method. The images obtained with HNAM were first Gaussian filtered before assessing the threshold value (Supplementary Fig. S2B), whereas the filtering was not required for images obtained with HRM (the same threshold value was found with and without filtering). The UTS values represent the quantity of voxels that cannot be uniquely assigned to the fiber or the matrix phase and are here presented as a percentage of the total number of pixels.
- *Gradient sharpness at the edges (GSE)* To quantify the sharpness at the edges, or inversely the blurriness, the maximum rate of change at each edge point was considered, which was given by the magnitude of the gradient (Supplementary Fig. S3). The GSE is the average edge gradient magnitude value normalized to the number of edge points. GSE was calculated from the sub-volumes containing only fat cells (Fig. 2D), primarily because the fat spheres have clear and sharp borders. Furthermore, the transition to the inter-fibre matrix can sometimes be unclear, which would induce large variability in the data.

#### Reproducibility

All analyses to determine image quality (Fig. 1I) were repeated in different sub-volumes of each sample moving along the tendon main axis three times for fixed and fresh samples and two times in embedded samples (sample shrinkage and cracks in the tissue reduced the volume available for the analysis).

#### Cross-sectional area

To quantify the level of tissue dehydration after fixation and embedding, the average cross-sectional area was calculated for the central ~ 500 µm of the tendons using MATLAB. The region was chosen in the middle of the tendon (equal distance from both bone and muscle) where the shape of the cross-section was the most consistent. However, it should be noted that even in the center of the sample the cross section can slightly change along the tendon length and fat is irregularly deposited along the outer sample. As the tendon tissue was not distinguishable from the background by contrast threshold (Supplementary Fig. S4A), the tendon cross section was selected after increasing the tissue contrast by projecting the maximum intensity at every 100 slices (Supplementary Fig. S4B). The contrast-enhanced cross section was binarized and the area was calculated (Supplementary Fig. S4C).

### Qualitative comparison of phase contrast enhanced tomography and histology

Two right Achilles tendons were harvested and incubated in the following solutions: 15% sucrose (Sigma Group, Malmö, Sverige, S0389) at 4 °C overnight, 30% sucrose at 4 °C overnight, a 1:1 mixture of optimal cutting temperature compound (OCT; VWR International AB, Spånga, Sweden, 00411243) and 30% sucrose for 20 min. They were then placed in 100% OCT, snap-frozen and stored at − 80 °C until sectioning. The tendons were sectioned longitudinally (7 µm thickness) comprising the full length of the tendon. One slide of each specimen was stained with hematoxylin and eosin and examined using an Olympus BX51 light microscope. The other slide was used for immunohistochemistry staining for collagen type 1 as follows. Directly after sectioning a blocking buffer containing 5% normal goat serum (Sigma Group, Malmö, Sverige; G9023) was added for 2 h. Primary polyclonal antibodies from rabbit were used (1:100, Abcam, Cambridge, UK; ab34710) and incubated for 18 h at 4 °C. A goat anti-rabbit IgG secondary antibody (1:400, Alexa Fluor Plus 594 Highly Cross-Absorbed Secondary Antibody; Thermo Fisher Scientific, Rockford, USA; A32740) was added for 2 h. The specimens were counterstained with DAPI (4′,6-Diamidino-2-Phenylindole Dihydrochloride; 1:2000; Thermo Fisher Scientific, Stockholm, Sweden; 62248) for 3 min and then mounted with ProLong gold antifade reagent (Thermo Fisher Scientific, Stockholm Sweden; P36930). The stained specimens were examined using a Lecia DMi8 microscope (Leica Microsystems, Wetzlar, Germany). The fluorescence was detected at 550 nm (collagen 1) and 385 nm (DAPI). The images were taken with a Hamamatsu Orca LT Flash sCMOS camera. The images were adjusted to the negative control staining, where the primary antibody was omitted, to correct for unspecific antibody detection. Kidney from rat was used as a positive control.

## Results

**Figure 3:**
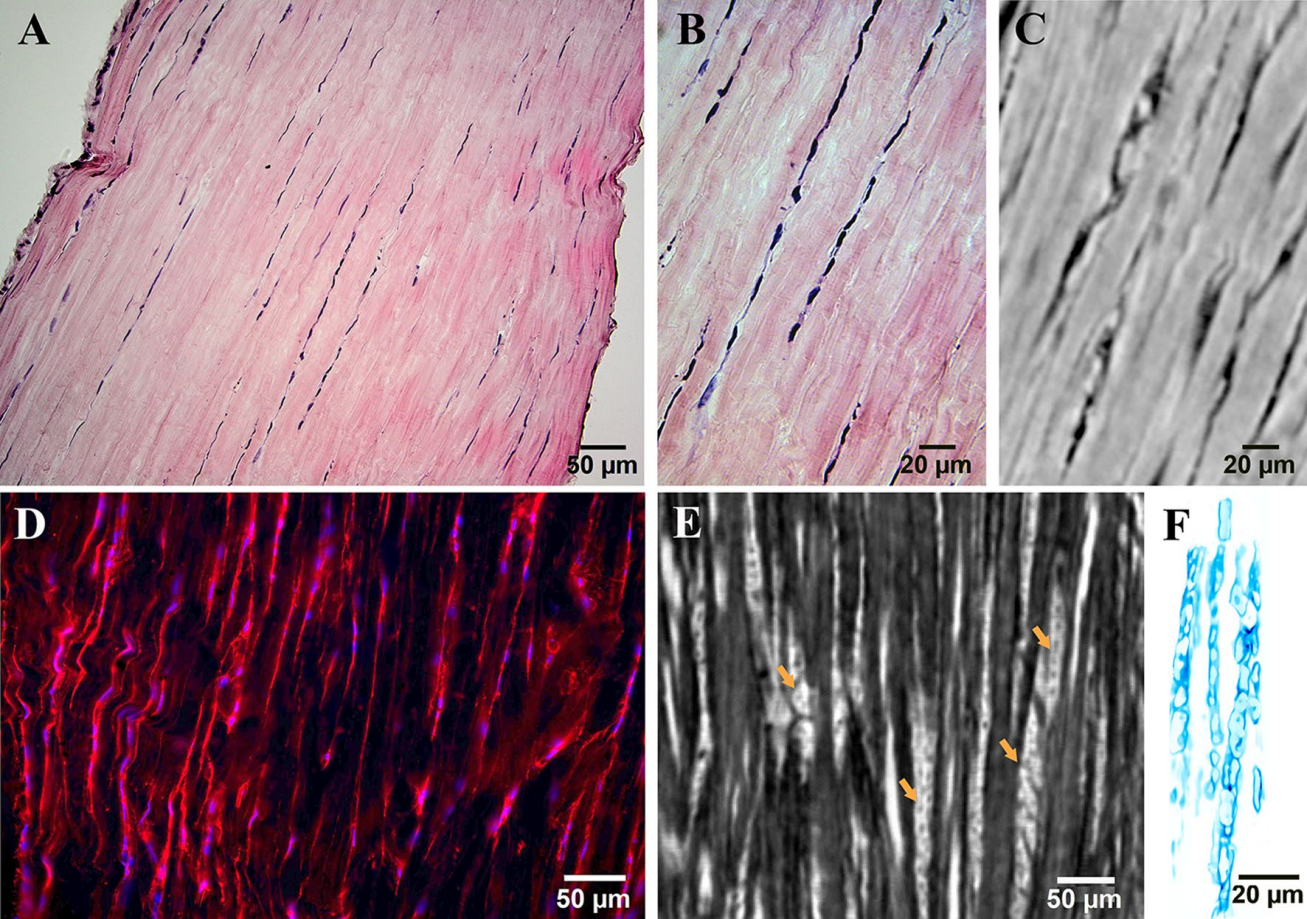
Comparison between fibers and cells observed by histology and by phase contrast enhanced tomography of fresh frozen samples in PBS. (**A**) Light microscope image (×20) of a histological slice stained with hematoxylin and eosin**;** (**B**) ×40 magnification of (**A**); (**C**) phase contrast enhanced tomographic slice showing fibers; (**D**) Fluorescent image (×25) of an immunohistochemistry slice stained for collagen I (red) and DAPI (blue); (**E**) cells (orange arrows) between the collagen fibers in a fresh sample imaged by phase contrast enhanced tomography. In (**E**), the contrast was inverted to increase the cell visibility (white) over fibers (black). (**F**) Volume rendering showing a few inter-fiber cells acquired by phase contrast enhanced tomography.

As this represents one of the first studies in which the tendon microstructure was imaged by phase contrast enhanced synchrotron tomography, we compared the tomographic data to state of the art histology to show that the internal tendon structure presents as expected (Fig. 3).

### The effects of sample preparation

**Figure 4:**
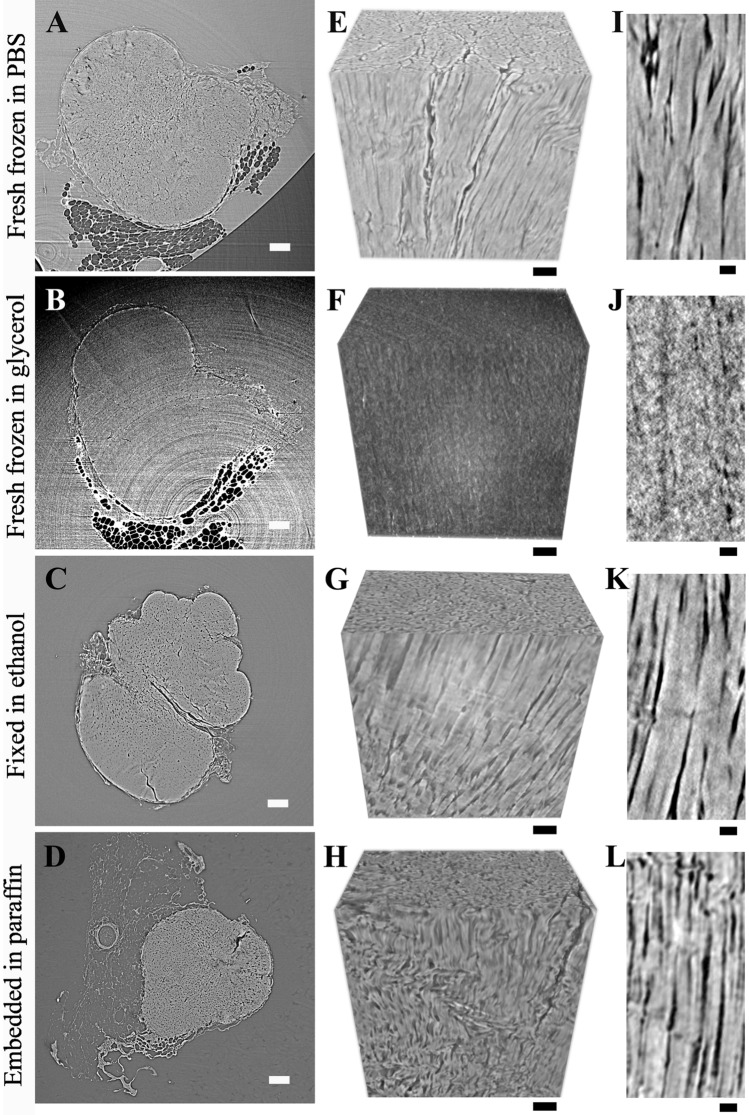
Sample preparation can affect the visibility of the internal structures. (**A**–**D**) Cross sections of the central region of the tendon for: (**A**) fresh frozen sample imaged in PBS, (**B**) fresh frozen sample in glycerol, (**C**) fixed tendon imaged in ethanol, (**D**) paraffin embedded sample. (**E**–**H**) 3D renderings of fibers in the central region of the respective tendons to the left, (**I**–**L**) longitudinal slice showing few fibers at high magnification. The darker region between fibers is the tendon non-collagenous matrix. All samples were imaged with HNAM. Scale bars: (**A**–**D**) 150 µm, (**E**–**H**) 50 µm, (**I**–**L**) 25 µm.

**Figure 5:**
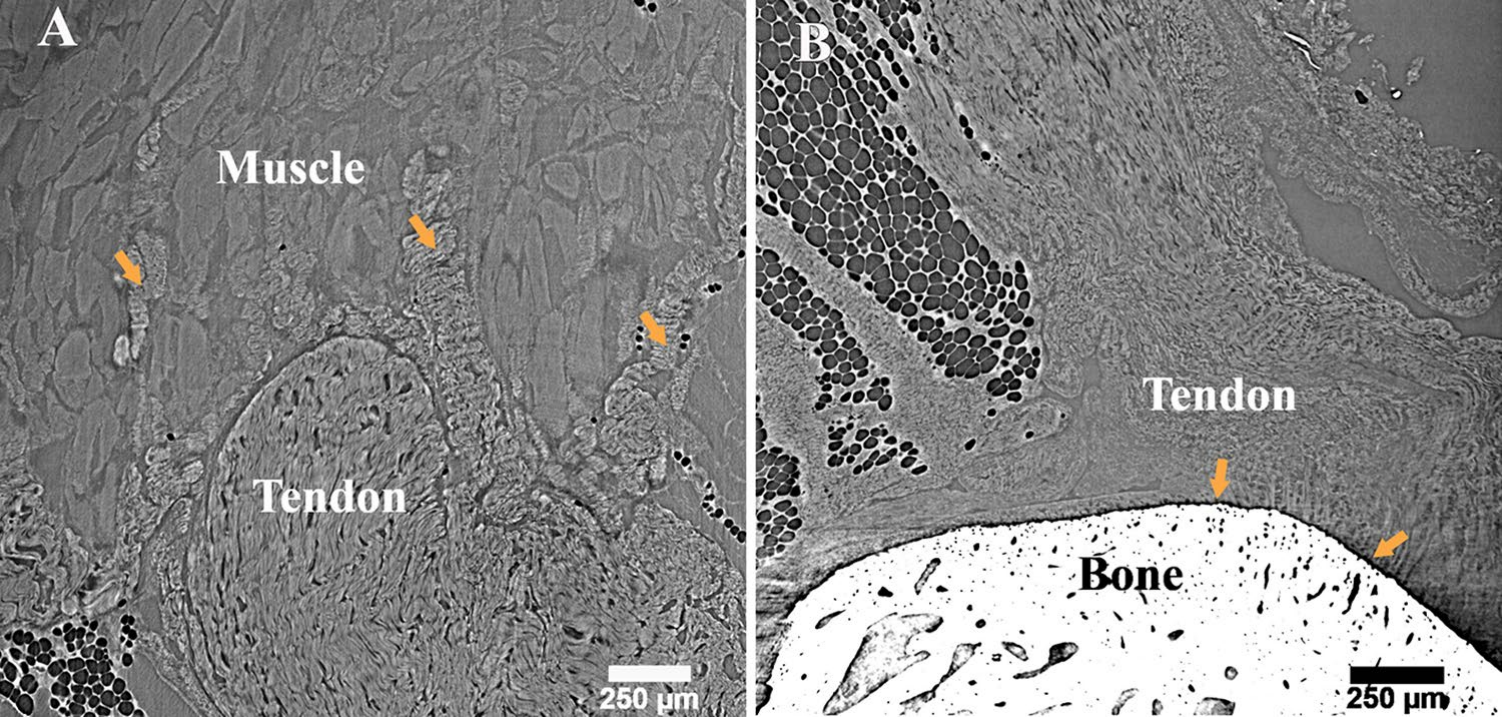
Fiber organization at the junctions. SR-PhC-μCT (HNAM) longitudinal views of: (**A**) The tendon–muscle junction showing tendon fiber intercalation into the muscles (orange arrows); (**B**) the tendon-bone junction characterized by a sharp passage from the tendon to the bone (orange arrows).

In the case of fresh frozen samples in PBS, fixed samples, and embedded tendons the internal fiber structure was visually distinguishable, and no image artifacts were clearly observed (Fig. 4). Only in the case of the fresh samples in glycerol, the contrast was reduced substantially by the medium so that the fibers were not visible and ring artefacts were clearly distinguishable (Fig. 4B,F,J). The fibers ran parallel along the tendon main longitudinal axes, and were tightly packed with only limited matrix being visible in between (Fig. 4). Furthermore, except for those immersed in glycerol, all sample preparations were suitable to study the tendon-bone and tendon–muscle junctions (Fig. 5A,B). As fibers closely intercalate into the muscular tissue, they presented a more crimped conformation than in the center of the tendon (Fig. 5A vs Fig. 4E,G,H). At the bone junction, the merging of the tendon into the bone was characterized by a sharp transition (Fig. 5B).

In fixed and embedded samples, cracks formed in the tissue (Supplementary Fig. S5) and the tendons shrank due to dehydration. In the central region of the tendons the average area of fresh samples in PBS was 1.39 ± 0.48 mm^2^, whereas for fixed samples and embedded samples the average areas were 1.02 ± 0.02 mm^2^ and 0.54 ± 0.40 mm^2^, respectively, indicating ~ 40% and ~ 60% reductions when compared to fresh samples. Fresh samples preserved a more pristine structure with cells being visible between the fibers (Fig. 3E,F, Supplementary video S2).

### Image quality

**Figure 6:**
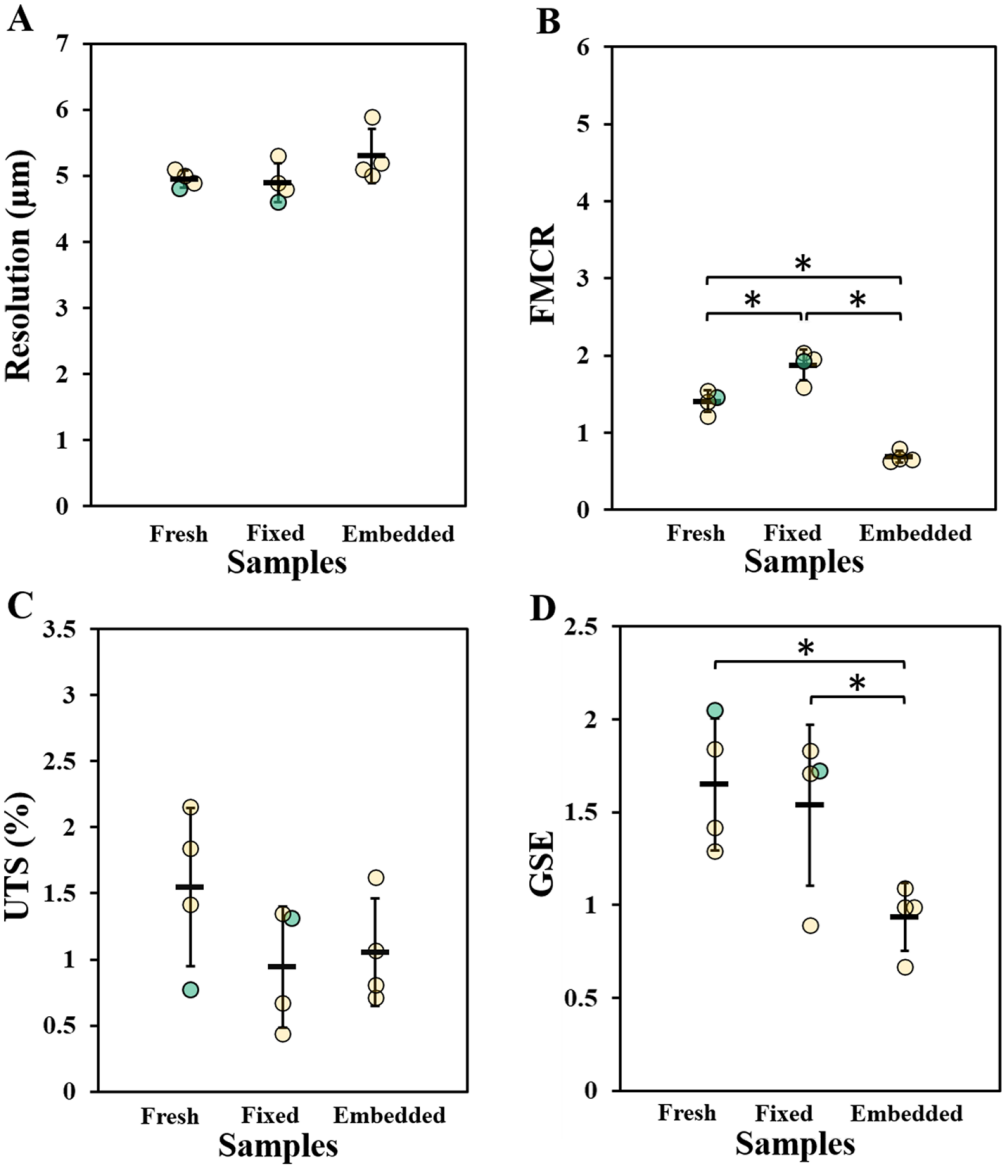
The image quality for different sample preparations. The quality of images acquired by HNAM was evaluated as: (**A**) spatial resolution, (**B**) fiber to matrix contrast ratio (FMCR), (**C**) uncertainty of the threshold-based segmentation (UTS), (**D**) magnitude gradient sharpness at the edges (GSE). Individual sample measurements are shown as circles, mean and standard deviation for each group are indicated. Fresh = fresh frozen samples in PBS, fixed = fixed samples in ethanol, embedded = fixed samples embedded in paraffin; samples that were also imaged with HRM are highlighted in green.

For fresh samples in glycerol, the image quality analysis was not conducted as it was not possible to distinguish the tendon internal structure due to e.g. low contrast and substantial ring artefacts. However, for the rest of the samples the resolution was only slightly affected by the type of preparation. The average resolutions were: 5.0 ± 0.1 µm (coefficient of variability, CV = 0.03), 4.9 ± 0.3 µm (CV = 0.06) and 5.3 ± 0.4 µm (CV = 0.08), for fresh, fixed, and embedded samples respectively (Fig. 6A). Differently from the resolution, the FMCR was affected by the sample preparation, where higher values correspond to better image quality. The FMCR was the highest for fixed samples and the lowest for embedded samples. The average FMCRs were 1.4 ± 0.1, 1.9 ± 0.2 and 0.7 ± 0.1 respectively for fresh, fixed, and embedded samples (Fig. 6B). The variability within the preparation groups was below 10% (CV ≤ 0.1 for all groups). The results further indicate that the segmentation uncertainty was comparable for fixed and embedded samples and slightly higher in the case of fresh samples (average UTS: 1.5 ± 0.6, 0.9 ± 0.5 and 1.1 ± 0.5 respectively for fresh, fixed, and embedded samples, Fig. 6C). However, for UTS there was a considerable variability between samples from the same preparation group (CV = 0.4, CV = 0.5, CV = 0.4 for fresh, fixed, and embedded samples respectively). The data showed that the edges were the least sharp (i.e. low GSE values) in the embedded samples and comparable for fresh and fixed samples (average GSE: 1.65 ± 0.36, 1.54 ± 0.44 and 0.94 ± 0.18 for fresh, fixed, and embedded samples respectively, Fig. 6D). Also for GSE, the intragroup differences were quite pronounced (CV = 0.3, CV = 0.2, CV = 0.2 respectively for fresh, fixed, and embedded samples). The four parameter values were consistent between sub-volumes of the same group (Supplementary Fig. S6).

### The effect of the imaging setup

**Figure 7:**
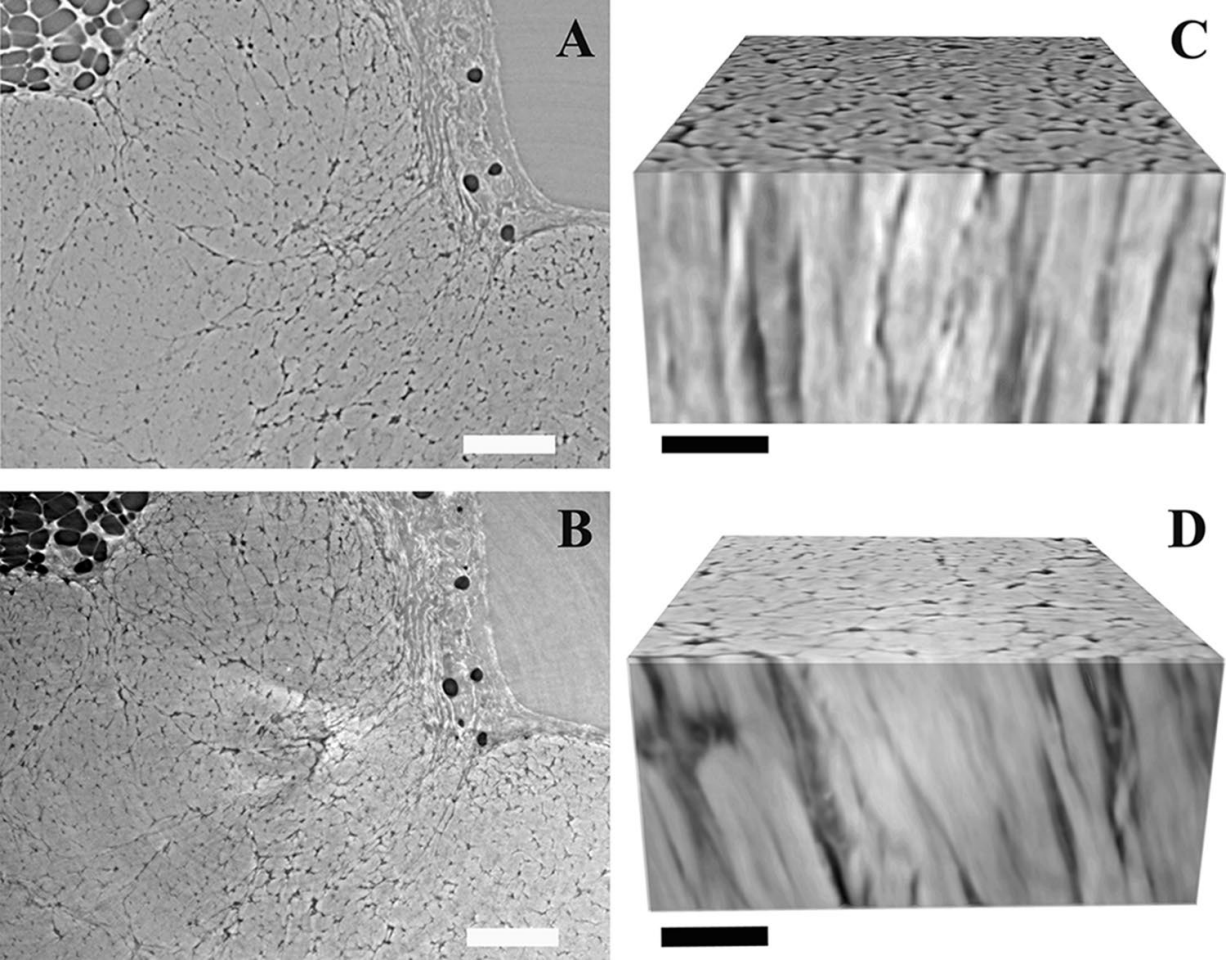
Tendon fibers visualization using two different imaging setups. Cross-sectional view of collagen fibers in the same sample imaged by (**A**) HNAM and (**B**) HRM. 3D renderings of the fibers imaged with (**C**) HNAM and (**D**) HRM. Scale bars: (**A**, **B**) 150 µm and (**C**, **D**) 50 µm.

By an initial visual inspection fiber contours were similarly distinguishable in images acquired by HNAM and HRM with no pronounced differences (Fig. 7).

**Figure 8:**
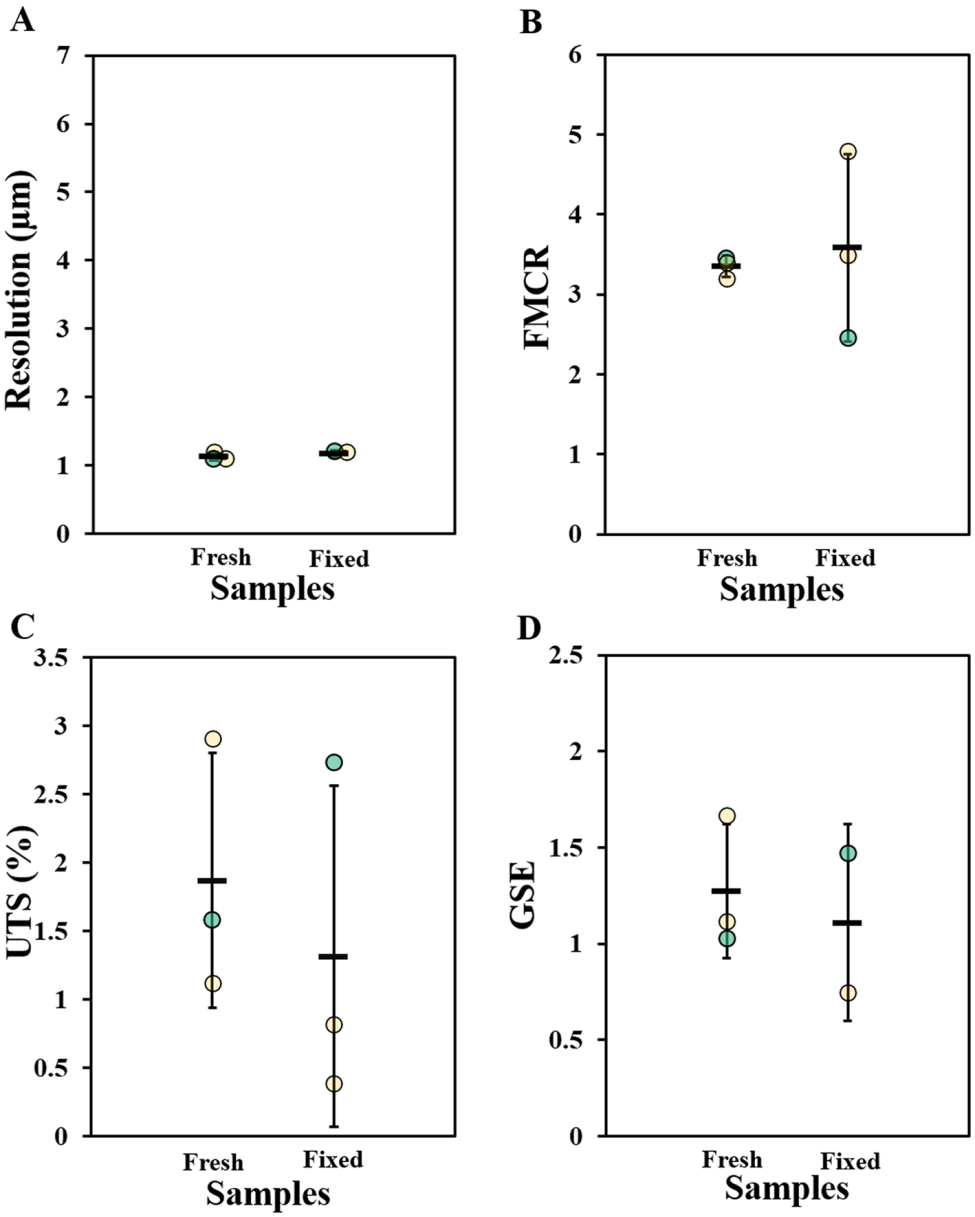
The image quality for the images acquired by HRM. The quality was determined by: (**A**) spatial resolution, (**B**) fiber to matrix contrast ratio (FMCR), (**C**) uncertainty of the threshold-based segmentation (UTS), (**D**) magnitude gradient sharpness at the edges (GSE), in this group n = 2 due to fact that in one sample no fat cells were visible in the FOV. Individual sample measurements are shown as circles, mean and standard deviation for each group are indicated. Fresh = fresh frozen samples in PBS and fixed = fixed samples in ethanol. Samples imaged with both microscopes are highlighted in green.

Imagining at five times higher magnification (HRM setup), the spatial resolution (1.2 ± 0.3 µm) was four times lower than for HNAM (4.6 ± 0.3 µm) (Fig. 6A vs Fig. 8A). The FMCR was higher for HRM compared to HNAM acquisitions, but not proportional to the increase in magnification. The contrast ratio was comparable for the two sample preparations (average FMCR: 3.4 ± 0.1 and 3.6 ± 1.2 for fresh and fixed samples, respectively, Fig. 8B). Imagining at five times higher magnification did not translate into a lower level of segmentation uncertainty (average UTS: 1.9 ± 0.9 and 1.3 ± 1.2 for fresh and fixed samples, respectively, Fig. 8C). Furthermore, the GSE did not substantially increase when HRM scans were considered (average GSE: 1.27 ± 0.35 and 1.11 ± 0.5 for fresh and fixed samples, Fig. 8D).

## Discussion

This work provides a comprehensive description of the Achilles tendon structure using phase-contrast enhanced synchrotron micro-tomography. Acquiring 3D high-quality images is fundamental to study complex biological structure and to enable precise segmentation of the structures of interest. Four types of sample preparations and two imaging setups were evaluated and compared. Fresh frozen, fixed, and embedded tissue enabled visualization of the tendon internal structure (Fig. 4). As imaging artefacts due to sample movements were reported previously for fresh frozen intervertebral disk samples^33^, we also investigated if a more viscous solution, such as glycerol, could help reduce possible micro-vibrations. However, this drastically reduced the image quality (Fig. 4B,F,J), resulting in the conclusion that glycerol solution is not suitable to study the tendon microstructure. The three other types of sample preparations were found suitable for the visualization of the tendon microstructure and junctions (Fig. 5A,B). Junctions are of considerable biological relevance since an understanding of how the tendon intercalates into the muscle and connects to the calcaneus bone could help rationalize how mechanical loading is transferred.

Our quantitative approach to determine the image quality showed that the spatial resolution was not substantially affected by the type of sample preparation (Fig. 6A). The level of precision of the segmentation of the fibers from the matrix was estimated by calculating FMCR and UTS. The FMCR was the highest for fixed samples and the lowest for embedded samples (Fig. 6B). We hypothesize that the increased contrast of the fixed samples compared to the fresh samples could be a consequence of the dehydration of the matrix due to fixation. Whereas, in the case of the embedded samples the lower FMCR could be attributed to high standard deviation as a consequence of apparent gray-value fluctuations as individual fibers are nearly resolved inside the bundles (Fig. 4L). Furthermore, due to sample dehydration, fibers did to some extent shrunk and more borders between adjacent fibers were visible compared with other sample preparations. In order to cover statistically relevant number of voxels the regions for FMCR calculations comprise often more than one fiber. An increased contrast between individual fibers therefore lowers the FMCR between interfascicular matrix and regions attributed to fibers (fiber bundles). Lovric et al. found that a contrast to noise ratio (FMCR in our case) of 2 was the lower limit for an effective segmentation of lung tissue^55^. We expected a similar limit to be sufficient to enable the segmentation of Achilles tendon fibers from the matrix, and this limit value was almost reached (for fixed samples, FTMCR = 1.9 ± 0.2). Furthermore, the uncertainty of the threshold segmentation was in all cases below 2.5% (Fig. 6B,C). Together these two factors indicated that the fibers could be distinguished from the matrix rather successfully for all sample preparations. However, further image processing could still improve the ability to segment the collagen fibers. In summary, the four considered properties showed similar trends for image quality, where lower resolution limit and UTS were associated with higher FMCR and GSE. Furthermore, the correlations between resolution and the three other properties consistently showed that the images of fresh frozen and fixed samples were characterized by similar quality, whereas the embedded samples had slightly inferior image properties (Supplementary Fig. S7). Lower quality images for embedded samples were also previously observed in the case of intervertebral disks^33^. However, it should be noted that for intervertebral disks the final image quality was further reduced by the presence of cracks in the paraffin blocks which were not observed in the embedded tendons.

Even though individual tendon fibers are harder to distinguish than when the sample undergoes cell maceration^40^, the fact that the imaging of fresh frozen samples in PBS solution resulted in high image quality is of great importance since this preparation allows visualizing the tendon structure as close as possible to the native conditions. This breaks ground for future research to study the relation between mechanical stimuli and structural response which would have not been possible in the case of more invasive sample preparations. In the future, the mechanical response of frozen-preserved samples could be studied by performing mechanical loading concurrently with synchrotron imaging (which has previously been done for e.g. bones on different length scales^56–58^). This would allow to directly determine how different fibers behave under mechanical stimulation. Furthermore, cells between fibers were visible (Fig. 3E,F). The morphologies and sizes of the observed cells strongly resembled the ones of cells shown in the histological slices (Fig. 3A,B) and the ones previously presented in other histological studies of tendons^11^. Tendon cells are responsible for synthesis and turnover of the collagen fibers and they respond to the local mechanical environment^59,60^. Consequently, to understand the relation between tendon structure and mechanical stimuli, the possibility of visualizing tendon cells in conditions close to native is significant. In addition to the advantages of studying a tissue in conditions close to native, when deciding on the most suitable sample preparation procedures, it should be considered that the ethanol used for fixation and embedding can cause shrinkage and cracking of the tendon tissue (Supplementary Fig. S5). The shrinkage of soft tissue caused by the dehydration process is a well-documented phenomenon which occurs due to the tissue processing steps during standard fixation and is not unique to Achilles tendons^41,61,62^.

After concluding that fresh and fixed samples showed similar image quality, we further investigated for these two sample preparations, if an increase in magnification would translate to a significant increase in image quality. The five times higher magnification setup (HRM) resulted in images with improved resolution and FMCR (Fig. 8A,B). The UTS and the GSE were not necessarily improved by the higher magnification, probably due to the presence of small regions with high noise levels (Fig. 8C,D). The four times improvement in resolution was substantial. However, the higher magnification comes with the drawback of more than six times higher radiation exposure. Accordingly, imaging Achilles tendons using the higher magnification setup could be advantageous for structural characterization. However, the HNAM setup seems more suitable for possible future studies of the relation between structure and mechanical loading, which requires well preserved tissue and careful planning to limit radiation dose^63–66^.

Despite the comprehensive design of the study, some limitations still exist. For instance, studying fresh samples, that had not earlier been frozen, would guarantee imaging the tissue in condition even closer to native. However, in our case it was not possible to scan the samples immediately after tendon dissection as the animal experiment and the synchrotron imaging were conducted in different countries. PBS and glycerol are widely used buffer solutions that help preserve samples and do not cause cell damage. However, they also do not chemically fix the samples. Therefore, the samples had to be frozen until just prior to the imaging to prevent degradation of the tissue. Furthermore, we did not consider imaging stained samples by laboratory X-ray tomography, which was previously proven successful for ligaments^67^. However, previous studies have also shown that, for similar types of non-mineralized biological samples, stain solutions such as PMA and PTA often do not fully penetrate the tissue^68–70^. Furthermore, even when the solutions penetrate (e.g. Logol’s iodine), the stained tissue does not always show more internal details or improved resolution compared to the unstained tissue^21,33^. Finally, for other types of biological samples such as muscle and heart tissues, recent methodological advances demonstrated that laboratory phase-contrast microtomography may in the future be implemented also for other types of soft tissues such as tendons^71,72^.

## Conclusions

This work demonstrates that SR-PhC-μCT imaging of fresh frozen Achilles tendons can provide a comprehensive anatomical understanding of the complex internal structure without invasive sample preparations. In Achilles tendons, structure and mechanical performance are strongly interconnected. Consequently, the possibility of studying samples in conditions close to their native state is of great relevance in the perspective of understanding the tendon microstructural response to mechanical loading. The results of this study and the proposed method to quantify image quality could be of use in future studies of other soft collagen based biological tissues (such as lungs, heart, and meniscus).

## Supplementary Information

Supplementary Information 1.

Supplementary Video S1.

Supplementary Video S2.
