# Supplementary material for: A quality optimization approach to image Achilles tendon microstructure by phase-contrast enhanced synchrotron micro-tomography
Source: Sci Rep. 2021 Aug 27;11:17313. doi: 10.1038/s41598-021-96589-w (PMC8397765; doi:10.1038/s41598-021-96589-w)
Supplement: Supplementary file 1 — Supplementary Information 1. [file 41598_2021_96589_MOESM1_ESM.docx]

**SUPPORTING INFORMATION**

**A quality optimization approach to image Achilles tendon micro-structure by phase-contrast enhanced synchrotron micro-tomography**

Maria Pierantoni, Isabella Silva Barreto, Malin Hammerman, Lissa Verhoeven, Elin Törnquist, Vladimir Novak, Rajmund Mokso, Pernilla Eliasson, Hanna Isaksson


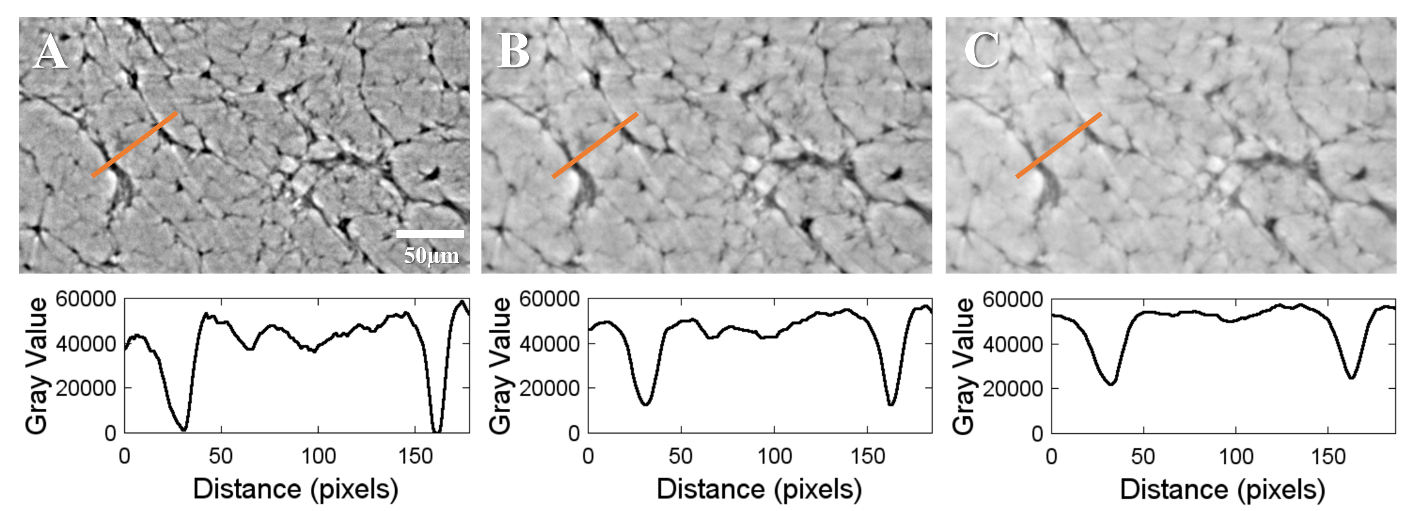


***Figure S1.*** ***Optimization of the ratio absorption coefficient to index decrement (β:δ) as function of the intensity profile*** *A) β:δ is 10:1, B) β:δ is 50:1, C) β:δ is 100:1. The ratio 50:1 was chosen to decrease the image noise whilst still keeping the image sharpness.*


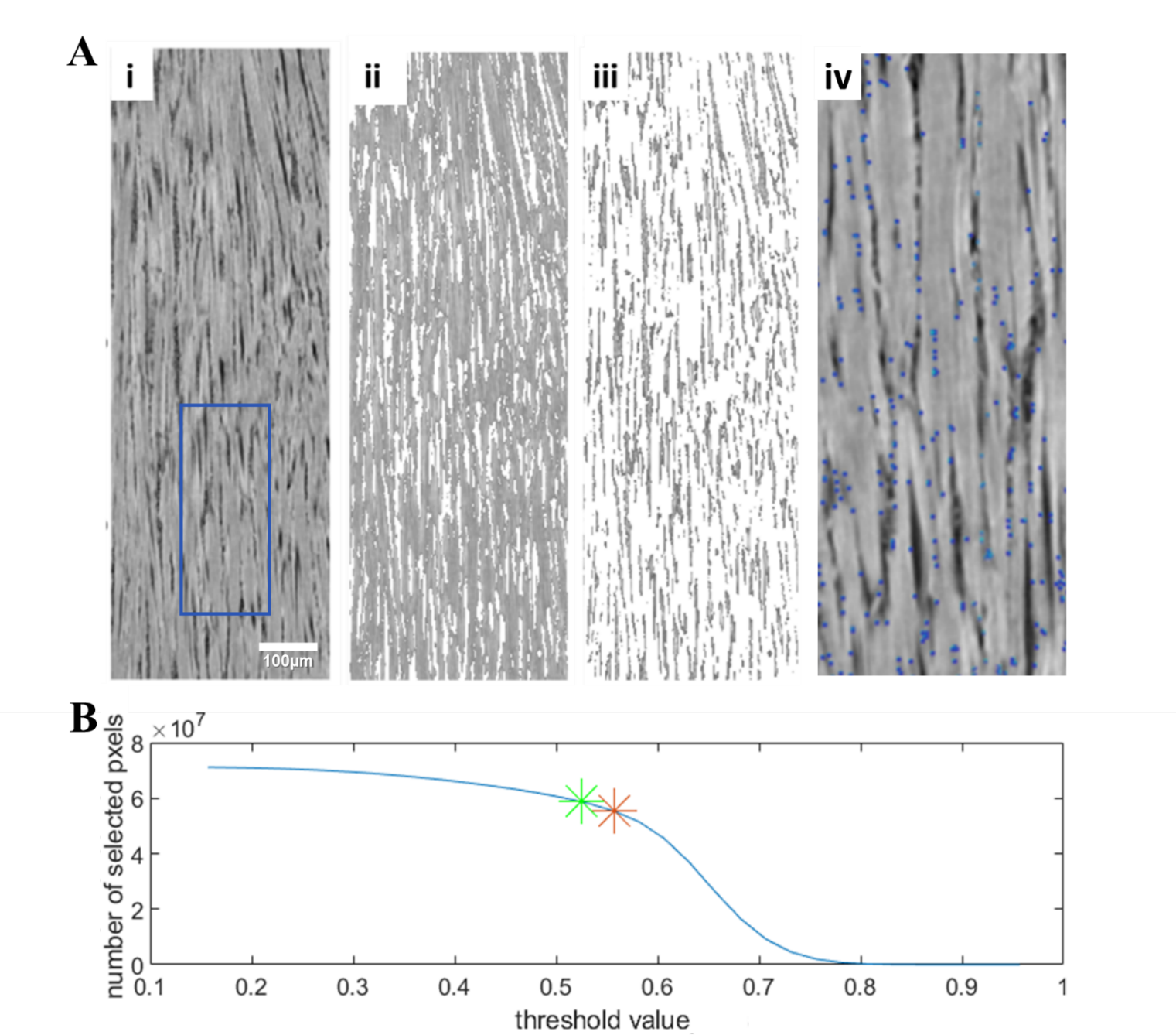


***Figure S2.*** ***Uncertainty of the threshold-based segmentation of fibers and matrix.*** *A) Selection of fibers and matrix: i) original image, ii) selection of the fibers, iii) selection of the matrix, iv) magnified region indicated by the rectangle in i), the blue dots represent the points at the borders between fibers and matrix where the tissue is selected both as fibers and as matrix. B) Dependence of the number of pixels selected as fibers on the threshold value. The value determined by Otsu’s method changes if a Gaussian filter is applied (orange asterisk: after filtering, green asterisk: without filtering).*


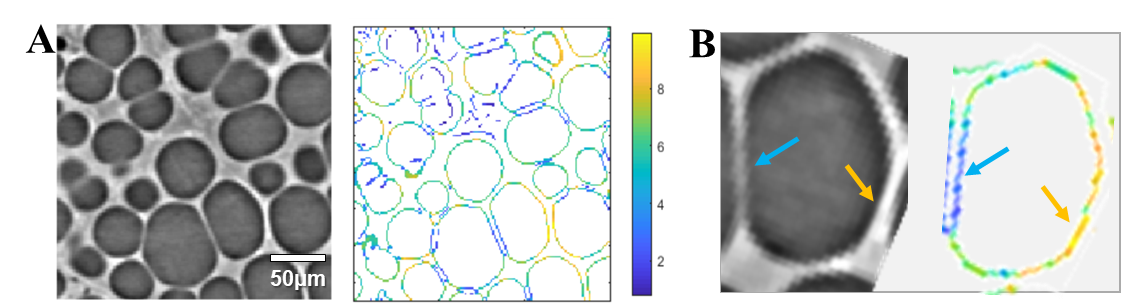


***Figure S3. A) Gradient sharpness at the edges*** *in the* *original image (left) and the magnitude gradient at the edges (right). B) Magnification showing that high GSE values are assigned to sharp edges and low values to blurred edges.*


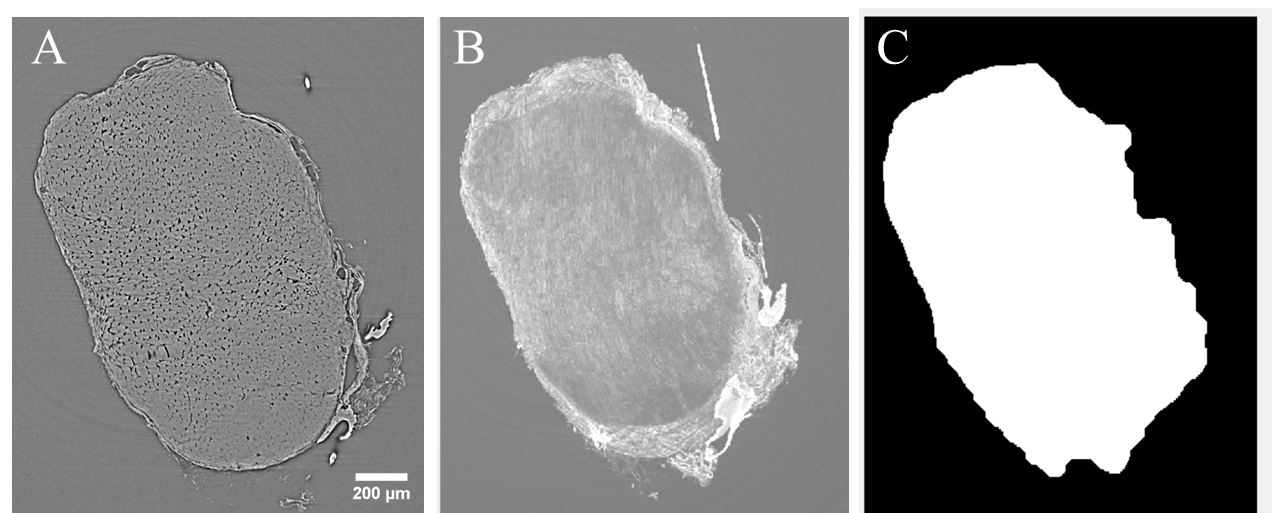


***Figure S4.*** ***Selection of tendon cross-sectional areas.*** *A) Original image, where the tendon tissue and the background cannot be distinguished simply by thresholding. B) Maximum intensity projection over 100 slices, where the tissue contrast is increased.* *The selection shows that the cross section can slightly change along the tendon length and that some fat along the sample is also selected (brighter region at the peripheries). C) Threshold-segmentation of the cross-section.*


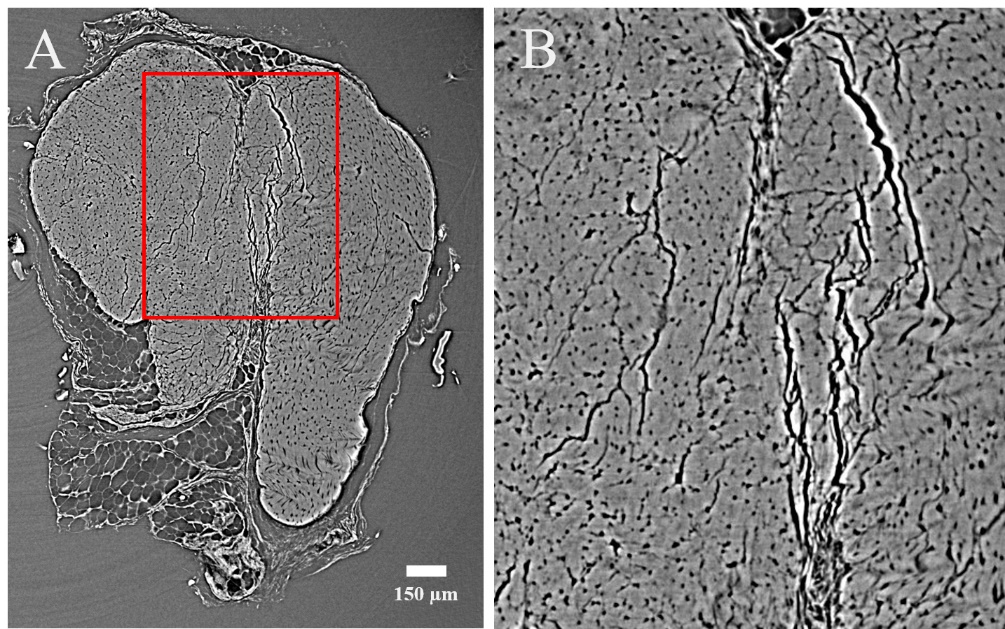


***Figure S5.*** ***Fixation dehydrates the tendon tissue and causes formation of cracks.*** *A) A fixed tendon cross-section showing diffuse cracking. B) Magnification of the area in the red rectangle in A).*


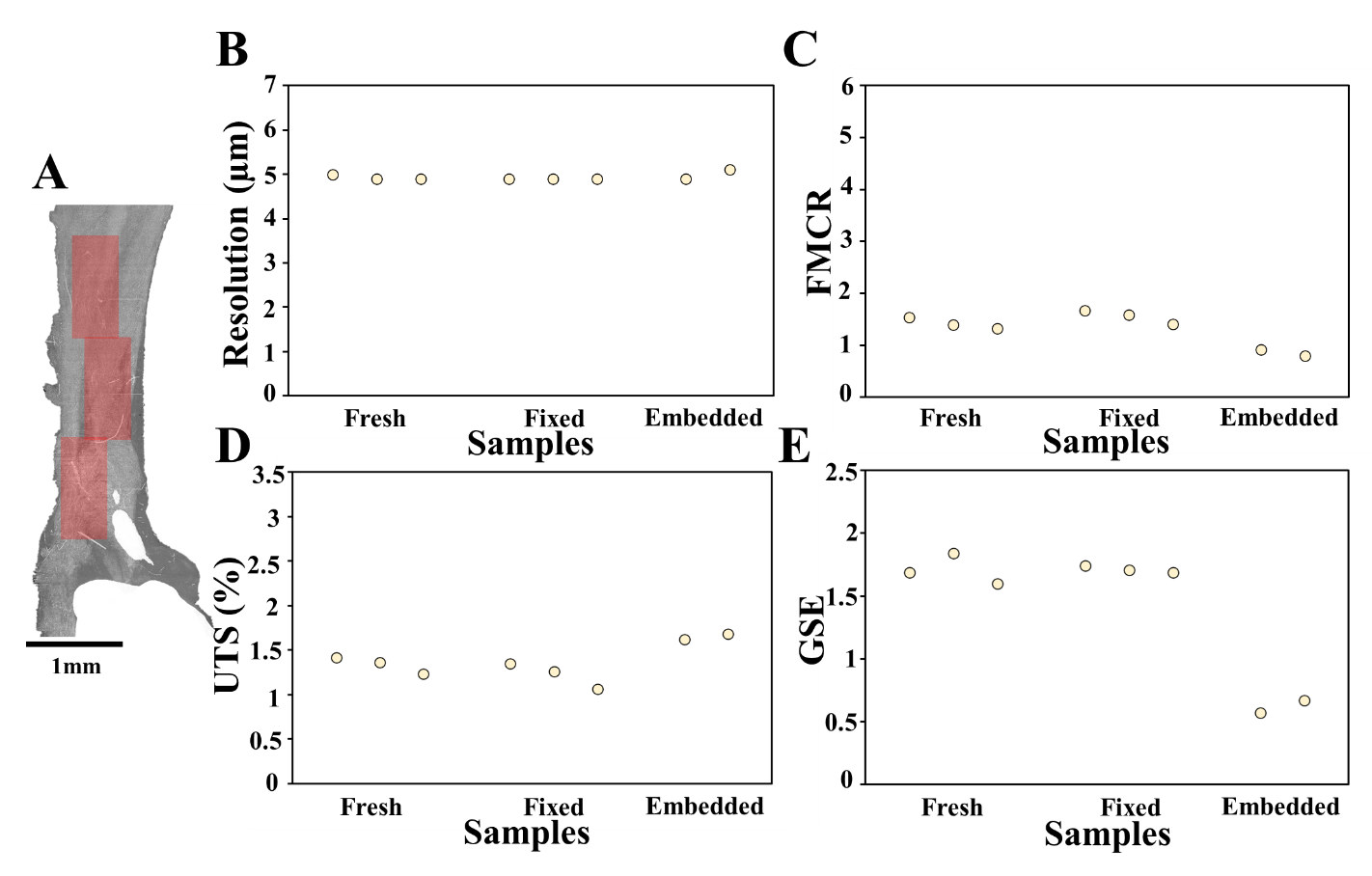


***Figure S6.*** ***The image quality is consistent among intra-sample regions.*** *A) Schematic representation of regions chosen for the analysis; B-E) Image analysis for different regions of one sample for each preparation: B) spatial resolution; C) fiber to matrix contrast ratio (FMCR), D) uncertainty of the threshold-based segmentation (UTS), and D) magnitude gradient sharpness at the edges (GSE).*


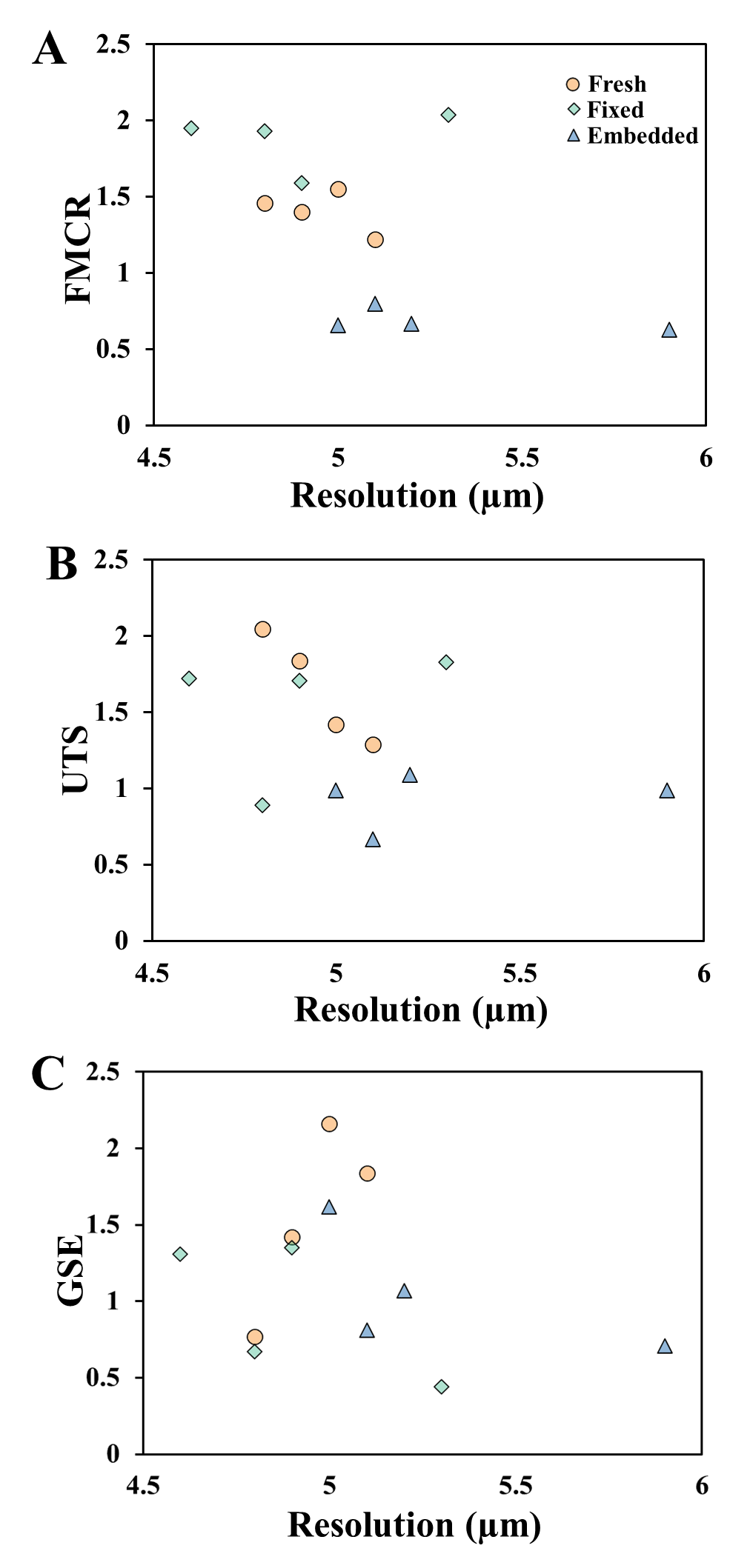


***Figure S7. Frozen and fixed samples show similar image quality and embedded samples have slightly inferior properties.*** *Correlations between resolution and: A)* *fiber to matrix contrast ratio (FMCR); B) uncertainty of the threshold-based segmentation (UTS); C) magnitude gradient sharpness at the edges (GSE). The samples showing the best properties are the one populating the top left side of the graph in A) and C) and the bottom left side of the graph in B).*

***Video S1. Representative sub-volume chosen for the image quality analysis.*** *The sub-volume is sliced along the tendon main axis showing the internal structure of the collagen fibers.*

*V****ideo S2. Cell structures between fibers****. The contrast was here inverted and adjusted so that the rounded cells between the fibers are now visible. The volume is sliced along the tendon main axis.*
